# Toward Large-Scale Energy Harvesting by a UV-Curable Organic-Coating-Based Triboelectric Nanogenerator

**DOI:** 10.3390/s23020579

**Published:** 2023-01-04

**Authors:** Jian Chen, Ning Tang, Li Cheng, Youbin Zheng

**Affiliations:** 1 Yangjiang Nuclear Power Company Ltd., Yangjiang 529941, China; 2 School of Electronic Information and Electrical Engineering, Shanghai Jiao Tong University, Shanghai 200240, China; 3 School of Materials and Energy, Lanzhou University, Lanzhou 730000, China; 4 Department of Chemical Engineering and Russell Berrie Nanotechnology Institute, Technion-Israel Institute of Technology, Haifa 3200003, Israel

**Keywords:** wearable devices, energy harvesting, triboelectric nanogenerator, organic coating

## Abstract

Triboelectric nanogenerators (TENGs) stand out as an attractive form of technology for the efficient harvest of mechanical energy and the powering of wearable devices due to their light weight, simplicity, high power density, and efficient vibration energy scavenging capabilities. However, the requirement for micro/nanostructures and/or complex and expensive instruments hinders their cheap mass production, thus limiting their practical applications. By using a simple, cost-effective, fast spray-coating process, we develop high-performance UV-curable triboelectric coatings for large-scale energy harvesting. The effect of different formulations and coating compositions on the triboelectric output is investigated to design triboelectric coatings with high output performance. The TENG based on a hybrid coating exhibits high output performance of 54.5 μA current, 1228.9 V voltage, 163.6 nC transferred charge and 3.51 mW output power. Moreover, the hybrid coatings show good long-term output stability. All the results indicate that the designed triboelectric coatings show great potential for large-scale energy harvesting with the advantages of cost-effectiveness, fast fabrication, easy mass production and long-term stability.

## 1. Introduction

The rapid development of flexible functional materials and advanced fabrication technologies has led to wearable devices being widely used in our daily lives, allowing us to monitor our health status and achieve individual eHealth [1,2,3]. Wearable devices require electricity to perform various functions, so the power source, typically conventional batteries, is an essential component. A significant increase in demand for sustainable and independent operation, lightweight, and flexibility has been seen with the development of wearable devices for health applications [4,5,6]. Conventional batteries, which are bulky and rigid, do not satisfy these requirements and will cause additional environmental burdens. Mechanical energy, the most widely distributed form of energy in the body, is the best source of energy when wearing wearable devices. A large number of body movements (e.g., running, walking, heart beating, breathing, talking, blinking, and swallowing) are performed every moment of every day, containing a large amount of biomechanical energy, which can be collected to power the wearable devices [7,8,9]. There are different types of energy harvesting devices that can convert mechanical energy into electricity, including the mechanisms of electromagnetic induction [10,11], piezoelectric effect [12,13], and triboelectric effect [14,15]. Taking the advantages of light weight, simple structure, high power density and efficient low-frequency vibration energy scavenging, triboelectric nanogenerators (TENGs) stand out as an attractive technology for efficient mechanical energy harvesting [16,17,18]. Based on the coupling effect of triboelectrification and electrostatic induction, TENGs can efficiently collect electricity from random, irregular, and/or low-frequency energy, such as mechanical vibration [19,20], wind [21,22], body motion [23,24], and ocean waves [25,26,27].

To push the TENGs into practical applications, many research advances have been made to improve the output performance of the devices, including surface modification [28,29], structure optimization [30,31], ion injection [32,33], and intermediate layer implantation [34,35], expanding the fields of application to self-powered sensing, smart wearables, and implantable electronics [36,37,38]. Despite these advances, most devices need micro/nanostructures and/or complex and expensive instrumentation, making inexpensive and large-scale mass production difficult, which has ultimately limited their practical applications. Developing new materials compatible with existing mass production techniques is urgent and meaningful to solve this problem. As a well-established industrial process, painting (mainly spraying, rolling, and brushing) is a versatile method for the mass production of films, showing great potential for the mass production of TENGs. In this respect, Chung and co-workers reported a superhydrophobic water-solid TENG, which was prepared by a commercial aerosol hydrophobic spray [39]. Later on, Yun and co-workers developed a commercial spray paint-based solid–solid TENG for smart traffic systems and security applications [40]. In addition to commercial spray, Liu and co-workers fabricated silk-fibroin based TENG by using a spray-coating process, which exhibits a maximum voltage of 213.9 V and power density of 68.0 mW/m^2^ [41]. Saqib and co-workers proposed a natural seagrass-based material for spray-coatable TENG [42]. Wang and co-workers fabricated new hydrophobic organic coatings for water-solid TENG and hydropower harvesting [43]. Kong and co-workers developed solid–solid coating TENGs with antiwear and healing properties [44]. By adding mesoporous silica and perfluorooctylethanol, this coating TENG reached the short-circuit current of 10 μA and the output voltage of 220 V. Although significant advancements have been achieved in paintable TENGs, few studies have been conducted to develop painting materials specially designed for high output TENGs.

In order to benefit the most from this energy-harvesting technology for wearable devices, we developed an organic coating that can be used for high-output TENGs via a simple painting process. Different formulations and coating compositions were evaluated to design high-performance triboelectric coatings. The TENG with 1:1 mixture of DFHMA and BA showed the highest output performance of 54.5 μA current, 1228.9 V voltage, 163.6 nC transferred charge, and 3.51 mW output power, as well as good long-term stability, and it can be used for large-scale energy harvesting through a fast and cost-effective spray-coating and a UV-curing process.

## 2. Materials and Methods

### 2.1. Materials

Trimethylolpropane triacrylate (TMPTA) and tripropylene glycol diacrylate (TPGDA) was obtained from Shanghai Guang Yi Chemical Co., Ltd. (Shanghai China). Methyl methacrylate (MMA) was obtained from Tianjin Zhiyuan Chemical Co., Ltd. (Tianjin, China). n-butyl acrylate (BA), 2-Hydroxy-2-methylpropiophenone (HMPP), and Phenylbis(2,4,6-trimethylbenzoyl)phosphineoxide (XBPO) were obtained from Shanghai Macklin Biochemical Technology Co., Ltd. (Shanghai, China). Dodecafluoroheptyl methacrylate (DFHMA) and 2,2,3,4,4,4-Hexafluorobutyl methacrylate (HFBMA) were obtained from Shanghai Qinba Chemical Co., Ltd. (Shanghai, China). Fluororesin was purchased from Shanghai Yitu Industrial Co., Ltd. (Shanghai, China). Nylon-11 was purchased from Tianjin Heowns Biochemical Technology Co., Ltd. (Tianjin, China). Polytetrafluoroethylene (PTFE) film, Kapton film, poly(ethylene terephthalate) (PET) film and copper foil tape were purchased from a local market. All other chemicals were of analytical grade and used without further treatment.

### 2.2. Preparation of the UV-Curable Triboelectric Coating

The UV-curable resin contains 80 wt % oligomers (HFBMA, DFHMA, Fluororesin, MMA, BA, 1:1 mixture of DFHMA and BA) and 20 wt % UV monomers (TPGDA and TMPTA with a weight ratio of 3:1). The coating solution was prepared by mixing the UV-curable resin with 3 wt % HMPP and 1 wt % XBPO in xylene. After spray-coating the as-prepared solutions onto the PET substrate, they were cured under UV light at room temperature for 3–5 min to obtain the triboelectric coatings. The coatings with different formulations are referred to as HFBMA, DFHMA, Fluororesin, MMA, BA, and DFHMA + BA, respectively.

### 2.3. Preparation of Nylon-11 Friction Layer

A mixture of 9.37 g dichloromethane, 8.63 g anhydrous formic acid and 2 g nylon-11 was mixed for 2 h at room temperature to obtain a clear solution. As substrates, Kapton films were cut into 4 cm × 4 cm and cleaned with ethanol, acetone, and deionized water. After that, a Nylon-11 solution was spin-coated onto Kapton films at 500 rpm for 5s and 3000 rpm for 30 s. Then, the Nylon-11/Kapton layers were annealed at 80 °C to evaporate residual solvents. Thereafter, copper foil tapes were attached to Kapton on the opposite side of Nylon-11 as the bottom electrode.

### 2.4. Preparation of the Coating TENG

To fabricate the coating TENGs, UV-curable triboelectric coatings with different formulations were prepared directly onto 4 cm × 4 cm copper foil tapes, working as one of the friction layers. To test the effects of the coating composition on triboelectric output, the mass ratio between the DFHMA oligomer and UV monomers was changed from 4:1 to 2:1, 1;1, 1:2, and 1:4. Afterwards, the UV-curable triboelectric coating and Nylon-11 friction layer were assembled into a triboelectric generator for output performance characterization.

### 2.5. Characterization

To investigate the output performance of the TENG, an IVCL17-56 motor was used to periodically press and release the device. Short circuit current is measured by SR570 low-noise current amplifier (Stanford Research System, Sunnyvale, CA, USA) and the output voltage is measured by NI 9215 (National Instruments, Austin, TX, USA). Data were collected using LabVIEW programs (National Instruments, Austin, TX, USA).

## 3. Results and Discussion

**Figure 1:**
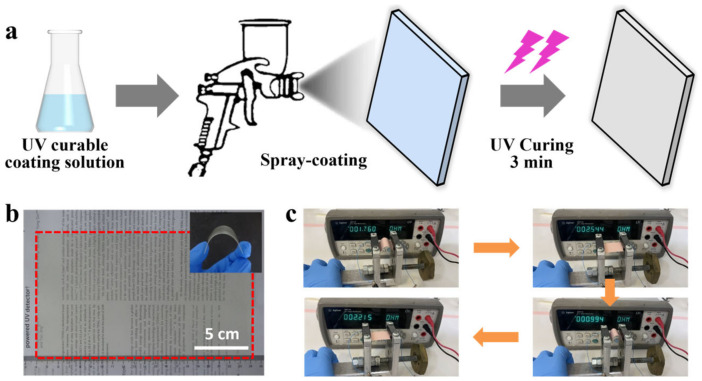
Fabrication process and flexibility of the triboelectric coating. (**a**) Fabrication of the triboelectric coating based on the spray-coating method. (**b**) The image of the large-area triboelectric coating on the PET substrate. The insert shows that the coating is flexible. (**c**) Resistance changes during repeated bending.

Among the polymers used in coatings, acrylic resins based on acrylate and methacrylate monomers dominate due to their excellent durability, weather resistance, gloss retention, adhesion, abrasion, and thermal resistance. So, in this work we chose four different acrylic monomers to develop an organic coating that can be used for triboelectricity harvesting. A commercial Fluororesin and PTFE film were also used as comparison. As mentioned in Section 2, UV-curable triboelectric coating solutions with different formulations were prepared and spray-coated onto the substrates, followed by UV curing for 3–5 min, as shown in Figure 1a. Spray coating is a simple, cost-effective, fast, and versatile process that can be applied to a wide range of surfaces, including flexible substrates. Further, this fabrication method can be easily extended to larger surfaces for mass production (Figure 1b). The inset in Figure 1b shows that our triboelectric coating is flexible after being sprayed onto PET film. As shown in Figure 1c, the triboelectric coating with copper foil tape exhibits stable mechanical flexibility during repeated bending and the resistance changed by less than 2 ohms. It is important to note that the coating solutions can be used not only for spray-coating, but also for other painting techniques, like brushing, rolling, and spin-coating, making it more practical.

**Figure 2:**
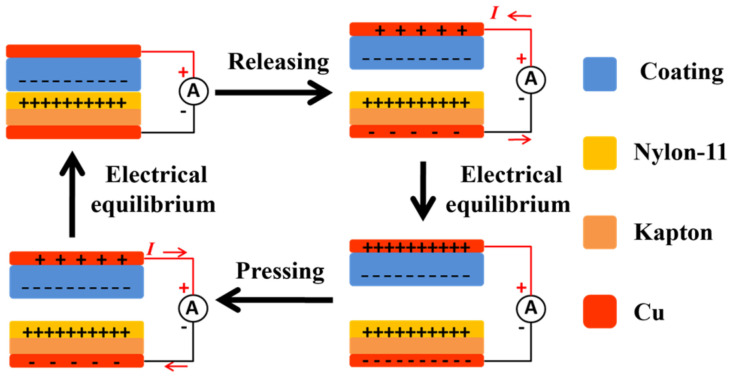
Schematic of the TENG’s electricity-generating process.

To obtain high output performance triboelectric coatings, we fabricated TENGs using triboelectric coatings with different formulations, including HFBMA, DFHMA, Fluororesin, MMA, BA, and DFHMA + BA. As a comparison, a commercial PTFE film based TENG was also fabricated. Nylon-11 spin-coated on Kapton film was used as another friction layer to construct TENGs. When the TENG was periodically pressed and released, Nylon-11 rubbed against the coating and generated positive charges on the Nylon-11 surface and negative charges on the surface of coating, according to the Triboelectric Series. As shown in Figure 2, when the TENG is periodically pressed and released, there is an alternating current generated between the top and bottom electrodes because of the coupling effect of triboelectrification and electrostatic induction. In this paper, the output performances of all the TENGs were tested under the same conditions of the working frequency of 5 Hz.

**Figure 3:**
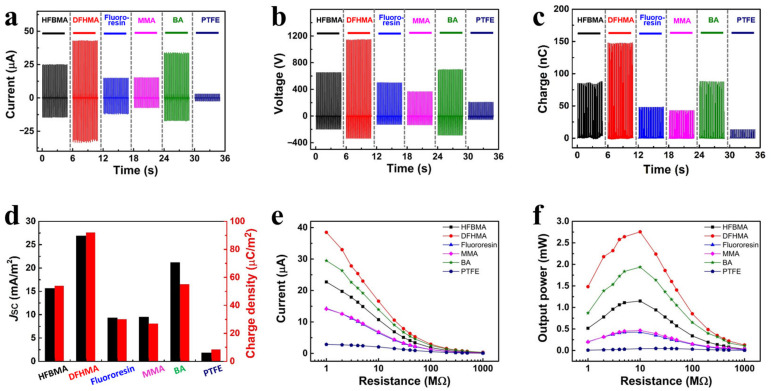
The output performance of the TENGs based on triboelectric coatings with different formulations and the commercial PTFE film: (**a**) short circuit current, (**b**) open circuit voltage, (**c**) transferred charge, (**d**) comparison by the bar plot of the current density and charge density. Current (**e**) and output power (**f**) of all TENGs with the different load resistance.

Figure 3a–c shows the short circuit current, output voltage and transferred charge of the TENGs based on triboelectric coatings with different formulations and the commercial PTFE film. With a current of 43.1 μA, voltage of 1148.5 V, and transferred charge of 148.3 nC, the TENG based on DFHMA clearly has the highest output performance. The TENG based on BA has the second highest output performance (34.1 μA, 698.4 V and 88.3 nC) and the lowest comes from the TENG based on the commercial PTFE film (3.0 μA, 207.2 V, and 13.6 nC). To further investigate the output performance, we calculated the current density and charge density for all the TENGs (Figure 3d). According to the results, the TENG based on DFHMA achieves a current density and charge density of 26.9 mA/m^2^ and 92.0 μC/m^2^, showing the same trend as short circuit current, output voltage and transferred charge. In order to determine the TENGs’ effective electric power, resistors are connected as external loads. The instantaneous current drops as the load resistance increases (Figure 3e) and the instantaneous output power reached its maximum value at a load resistance of 10 MΩ (Figure 3f). The output power of the TENG based on DFHMA, BA and commercial PTFE film is 2.76, 1.94, and 0.04 mW, respectively. Together these results indicate that the TENG based on DFHMA has the best output performance, which offers 69 times more output power than the TENG based on the commercial PTFE film.

**Figure 4:**
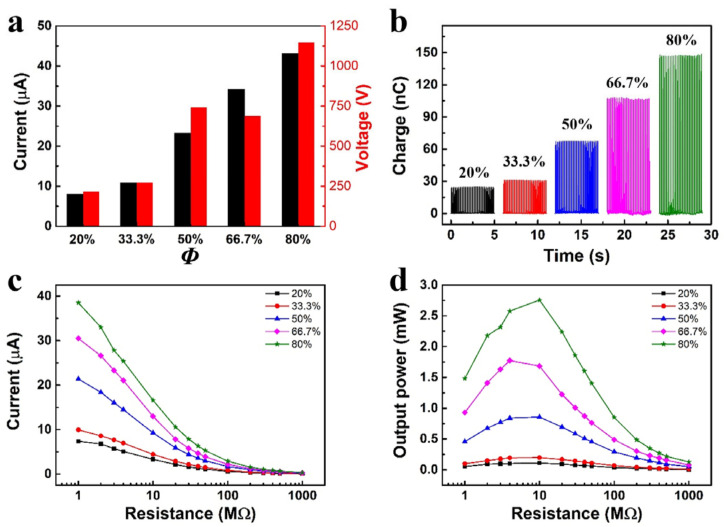
The coating composition as a function of the triboelectric output. (**a**) Bar plot of the short circuit current and open circuit voltage, (**b**) transferred charge. Current (**c**) and output power (**d**) of the TENGs with different *Φ* under the different load resistance.

Along with coating formulations, we also examined the coating composition in relation to the triboelectric output. Specifically, the mass ratio between DFHMA oligomers and UV monomers was modulated as 4:1 to 2:1, 1:1, 1:2, and 1:4. To simplify the discussion, the composition of triboelectric coating is defined as *Φ* DFHMA oligomers + (1 − *Φ*) UV monomers, where *Φ* is the mass ratio of DFHMA oligomers to UV monomers. TENG output performance with different *Φ* was evaluated and shown in Figure 4a,b. The current, voltage, and transferred charge all showed the same trend and increased with *Φ*. This is primarily because DFHMA oligomers contain a large number of fluorine groups, while fluorine is the most electronegative element due to its strong ability to attract electrons. Increasing means adding more fluorine, which is beneficial to the output performance. This is also confirmed by the results of TENGs’ effective electric power (Figure 4c,d). It can be seen that the output power of the TENG with *Φ* = 80% is the highest, which is consistent with previous results.

**Figure 5:**
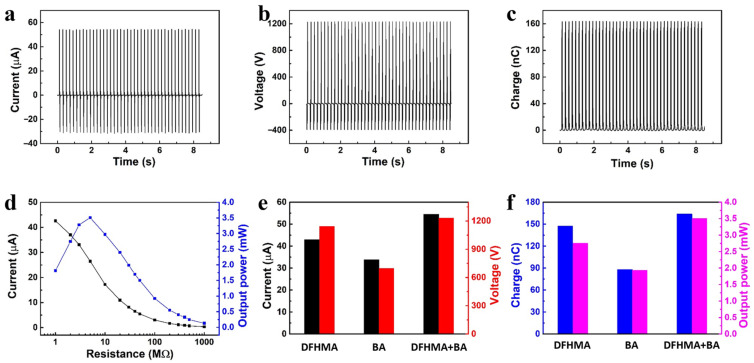
The output performance of the TENG based on hybrid compositions. (**a**) short circuit current, (**b**) open circuit voltage, (**c**) transferred charge, (**d**) current and output power under the different load resistance. Comparison by bar plot of the current and voltage (**e**) and transferred charge and output power (**f**) of the TENGs based on DFHMA, BA and 1:1 mixture of DFHMA and BA.

As part of our efforts to design a high-output triboelectric coating, we investigated how mixing two components would affect its performance. A TENG with a 1:1 mixture of DFHMA and BA was fabricated and examined. As shown in Figure 5a–d, the TENG based on hybrid compositions has very high output performance of 54.5 μA current, 1228.9 V voltage, 163.6 nC transferred charge and 3.51 mW output power. It is very interesting that hybrid composition coatings have higher output performance than single composition coatings (Figure 5e,f). It is particularly noteworthy that the TENG based on hybrid compositions has an output power approximately 87 times higher than the TENG based on a commercial PTFE film. Table 1 shows the comparison of output performances of TENGs based on a spray-coating process with our acrylic resin-based hybrid coating TENG. This finding provides an exciting avenue to optimize the output performance of coating based TENGs.

**Figure 6:**
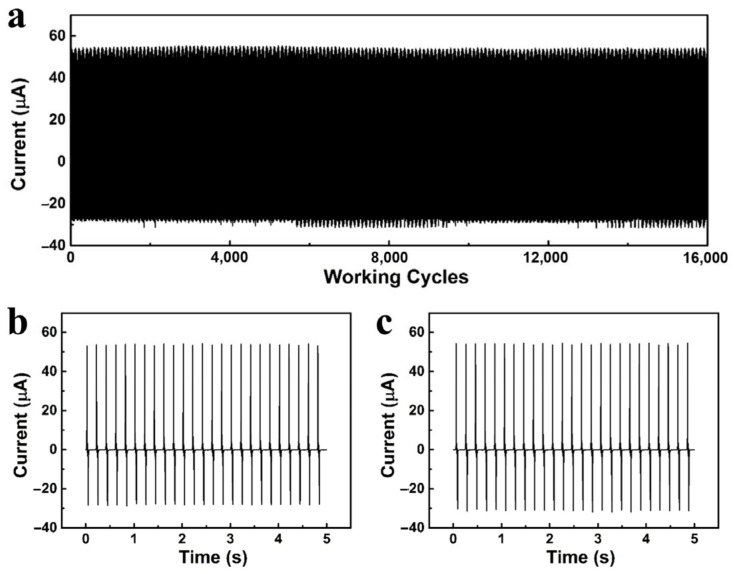
The mechanical stability of the TENG based on hybrid compositions: (**a**) the current for 16,000 working cycles, (**b**) the current for the beginning point, (**c**) the current for the ending point.

As a triboelectric coating, robustness plays a crucial role in practical applications besides outputting high performance. To fully evaluate our triboelectric coating, we carried out a long-term output stability experiment for the TENG with hybrid composition coatings. As shown in Figure 6a, the TENG with hybrid composition coatings exhibits very good mechanical stability even after more than 16,000 working cycles. The current does not decrease between the starting and ending points (Figure 6b,c), showing that our triboelectric coating has great potential for long-term large-scale energy harvesting.

## 4. Conclusions

In summary, we produced high-performance triboelectric coatings by using a simple, cost-effective, fast spray-coating process. In addition, the effect of different formulations and coating compositions on the triboelectric output was fully investigated. The hybrid composition coatings showed the best output performance as well as good long-term output stability. The hybrid composition-based TENG exhibited an output power approximately 87 times higher than that of the commercial PTFE film-based TENG, which opens up an exciting opportunity to improve the output performance of coating-based TENGs towards large-scale energy harvesting.

## Figures and Tables

**Table 1:** Comparison of output performances of TENGs based on spray-coating process.

Materials	TENG Type	Output Voltage (V)	Output Current (μA)	Power/Power Density	Reference
Natural seagrass	Solid–solid	288	40	1690 μW/70.42 μW/cm^2^	[42]
Silk-fibroin	Solid–solid	213.9	N/A	68.0 mW/m^2^	[41]
Commercial spray paint	Solid–solid	80	7.2	17.60 mW/m^2^	[40]
Mesoporous silica and perfluorooctylethanol loaded acrylate coating	Solid–solid	220	10	820 μW	[44]
Commercial aerosol hydrophobic spray	Water-Solid	13.4	2.1	N/A	[39]
Fluorine-modified acrylic resin coating	Water-Solid	4	4	2.88 μW/2.83 mW/m^2^	[43]
Fluorinated polyacrylate resin modified polyacrylic acid coating	Water-Solid	32	20	N/A	[45]
Acrylic resin-based hybrid coating	Solid–solid	1228.9	54.5	3.51 mW	This work

## Data Availability

The experimental data presented in the present paper are available from the corresponding author upon request.
